# Health‐related Quality of Life in People living with Dementia – Over and Underestimation of Proxy‐ratings

**DOI:** 10.1002/alz.089782

**Published:** 2025-01-09

**Authors:** Maresa Buchholz, Lidia Engel, Niklas Weber, Bernhard Michalowsky

**Affiliations:** ^1^ German Center for Neurodegenerative Diseases e.V. (DZNE), Site Rostock/Greifswald, Greifswald Germany; ^2^ Monash University, Melbourne, VIC Australia

## Abstract

**Background:**

Proxy ratings primarily provided by informal caregivers are usually administered if patients living with dementia (PlwD) are cognitively unable to rate health independently. The literature is limited by the use of typically agreement statistics, reporting that proxies generally underestimate PlwD health. Additional analyses of self‐ and proxy‐rated discrepancies in individual responses that focus on HRQoL dimensions are lacking. We examined discrepancies between self‐ and proxy‐rated health in PlwD on an individual response level.

**Method:**

We analyzed baseline data of n = 174 dyads (PlwD and their caregivers) of a cluster‐randomized, controlled intervention trial. HRQoL was assessed with the EQ‐5D‐5L from a self‐ and proxy‐proxy perspective together with sociodemographic and clinical data. The EQ‐5D‐5L consists of five dimensions (“mobility”, “self‐care”, “usual activity”, “pain/discomfort”, “anxiety/depression”), each with five response options. Initially, we calculated agreement statistics (weighted kappa) for each dimension. For individual response levels, we analyzed the degree of inconsistencies between self‐ and proxy ratings ranging from 0 (identical responses in all dimensions) to 5 (different responses in all five dimensions). We grouped the PlwD into “no problems” and “problems” depending on the EQ‐5D‐5L response of each dimensions to compare the response distances between PlwD and caregivers.

**Result:**

PlwD had a mean age of 80.1 years (49.1% female) with a MMSE mean score of 19.2; informal caregivers were, on average, 67.9 years old (67.8% female). Every third PlwD lived alone. In general, PlwD rated their HRQoL better than proxies (EQ‐5D‐5L indices: 0.75±0.25 vs 0.68±0.23; p<0.01). Nine dyads (5%) showed identical ratings, whereas 95% rated their health differently. Weighted kappa coefficients were lowest for the dimension usual activity (0.23) and highest for pain/discomfort (0.40). Among the grouped PlwD without and with problems, proxies estimated PlwD HRQoL worse (underestimation) or better (overestimation), respectively. This was due to a central‐tendency‐bias of caregivers, two times more frequently selecting mid‐response options, particularly apparent in mobility, usual activities, and self‐care.

**Conclusion:**

The two trends of contrary response behaviour (over‐ and underestimation & central tendency bias) could be caused by uncertainties of caregivers, underlining the challenge of using both perspectives interchangeably. Further research is needed when using HRQoL measures as proxy instruments.

